# Pathological Findings in Cattle Slaughtered in Northeastern Algeria and Associated Risk Factors

**DOI:** 10.3390/vetsci9070330

**Published:** 2022-06-30

**Authors:** Nora Mimoune, Mourad Hamiroune, Said Boukhechem, Choayb Mecherouk, Khaled Harhoura, Djamel Khelef, Rachid Kaidi

**Affiliations:** 1 Clinical Department, Animal Health and Production Laboratory, National High School of Veterinary Medicine, Algiers 16004, Algeria; n.mimoune@ensv.dz (N.M.); abbas.zrai.olives20@gmail.com (K.H.); djamelkhelef@yahoo.fr (D.K.); 2 Department of Agro-Veterinary Sciences, Faculty of Nature and Life Sciences, Ziane Achour Djelfa University, Route Moudjbara, PB 3117, Djelfa 17000, Algeria; 3 Institute of Veterinary Sciences, University of Mentouri Constantine 1, PB 325, Constantine 25017, Algeria; boukhechems@hotmail.fr; 4 Institute of Veterinary Sciences, Saad Dahleb University, Blida 09000, Algeria; noradoct@yahoo.fr (C.M.); kaidirachid84@gmail.com (R.K.)

**Keywords:** abattoir, lesions, incidence, sex, race, age, public health

## Abstract

**Simple Summary:**

Meat constitutes a very favorable ground for infectious or non-infectious diseases that can lead to economic losses and can also constitute a serious risk on public health. In this study, we determined the prevalence of the bovine diseases in a slaughterhouse in northeastern Algeria and the associated risk factors. The results showed a high frequency of animals presenting different lesions. The highest rate of pathological findings was observed on the liver followed by the lungs, whereas the lowest rate was recorded on the digestive system followed by the kidneys. In addition, the liver and lungs were more contaminated with hydatid cyst compared to other organs. Our data showed that females were more affected than males. Furthermore, young cattle from the local breed were the most affected. Our data confirm the importance of the bovine diseases requiring research in Algerian slaughterhouses and testify the real risk represented by the consumption of organs affected by these lesions. Therefore, it is crucial to implement an extension and control program in this region depending on the epidemiological aspect of the lesions.

**Abstract:**

Meat is a food of animal origin, which can be contaminated by infectious, parasitic and other non-infectious agents responsible for diseases, which threaten the health of consumers. This still poses a public health problem in Algeria and in many countries. In order to assess the epidemiological situation of certain diseases in the Taher region in Jijel and to determine the influence of certain variation factors and to estimate the risk on public health, a study was extended over a period of 14 months on a total of 1756 cattle slaughtered at the Taher slaughterhouse. The results showed that 609 cattle (34.68%) showed lesions. The highest rate of pathological findings was observed on the liver (37.27%) followed by the lungs (30.21%). The lowest rate was recorded on the digestive system (0.33%) followed by the kidneys (1.14%). In addition, the liver and lungs were more contaminated with hydatid cyst compared to other organs (20.69%, 19.05%, respectively). Our data showed that the diseases affected more females (55.82%) than males (44.17%) (*p* < 0.001). Furthermore, cattle aged between 3 and 5 years were the most affected (43.51%) and local breed cattle showed more lesions (71.59%). These results testify to the real risk represented by the consumption of organs affected by diseases, and the need to recognize the agents of contamination and the mode of transmission and to implement an extension and control program in this region depending on the epidemiological aspect of the lesions.

## 1. Introduction

Meat is the most valuable livestock product and, for many people, serves as a prime source of animal protein. It is either consumed as a component of kitchen-style food preparations or as processed meat products [1]. It constitutes a very favorable ground for parasitic diseases, such as fasciolosis and hydatidosis, infections, such as tuberculosis and abscesses, and physical issues, such as traumatic meats, which are zoonotic or non-zoonotic pathological findings with the mode of transmission either direct or indirect and causing serious economic losses.

The Algerian national production of red meats stood at 5.44 million quintals (qs) in 2017 for a value of 596 billion DA. By category, production was 3.25 million qs of sheep meat, 1.25 million qs of beef, 0.42 million qs of goat meat, 0.1 million qs of camel meat and 141 qs of horsemeat [2]. According to the data, the country’s imports of red bovine meats reached 122 million $ for the first ten months of 2020 alone, including 67.5 million $ in fresh meat and 54.5 million $ in frozen meat. Thus, there was an upward trend in these imports despite the availability of the local product, which is against the national economy [3].

On the other hand, meats and offal can be the seat of several foodborne infections caused by infectious agents which can constitute a major risk to the public health [4]. Most contamination of endogenous origin is of respiratory and intestinal origin during the evisceration and cutting of carcasses. Infectious agents are responsible for pathologies such as septicemic, abscess and pneumonia. In the case of deep abscesses, the presence of germs such as *Staphylococci aureus* is responsible for food poisoning when consuming semi-cooked grilled meats. Likewise, staph toxins are heat resistant and able to persist, especially in semi-cooked ground meat. In addition, during sepsis, the presence of germs such as *Salmonella* that contaminate meats are responsible for food poisoning when eating half-cooked grilled meats. This requires compliance with the rules of hygiene, handling and preparation of meat. Livestock may in addition act as reservoirs for pathogens that can also infect humans, a particular problem where humans and farmed animals come into close contact [1]. Besides, veterinary treatments, mainly antibiotics, used for therapeutic or prophylactic purposes in dairy cows [5], may be the cause of the presence of their residues in milk and meat. These residues can sometimes be a danger for the consumer by triggering rare allergic and toxic cases, or by promoting the emergence of a multi-resistant microflora [6]. Meanwhile, consumer expectations of food are changing, with increasing questioning of the quality of what is being offered by current agriculture, including animal production [7].

The main objective of this study was to explore the prevalence of certain diseases requiring research in the Taher slaughterhouse in Jijel, the health risks that could be caused and finally the variation factors involved in the incidence of these diseases. This work aims to propose the corrective measures necessary to fight against the sources of transmission of these pathological findings in order to preserve public health.

## 2. Materials and Methods

### 2.1. Study Area

The center of the region of Jijel is located 350 km east of Algiers. Its surface is 2398 km. It is located in the northeast of the country, on the Mediterranean coast. It is limited to the west by the region of Bejaia, to the south by Constantine and Mila, to the north by the Mediterranean Sea and to the east by Skikda [8]. The commune of Taher is located 15 km east of the city of Jijel, which has a population of 77,367 since the last census (RPGH) in 2008. It is bounded: to the north by the Mediterranean Sea; in the south by Chahna and Oudjana; to the east by the commune of Chekfa and Chahna; and in the west by the municipality of Emir Abdel Kadar [9].

### 2.2. Animals and Study Method

#### 2.2.1. Animals

A total of 1756 animals were slaughtered and examined for detection and suspicion of certain pathologies in the slaughterhouse in the Taher commune in the Jijel region over a period of 14 months. Data from 1756 cattle were collected.

The slaughter of animals was carried out from Saturday until Wednesday and began at eight in the morning until noon. Two veterinary inspectors carried out health inspections.

#### 2.2.2. Ante-Mortem Examination

It was carried out on live animals, before slaughter. Its objectives are to identify animals showing signs of disease throughout the herd, in order to give an early idea of suspicious pathological findings. The animals must be submitted to a water diet and sufficient rest.

#### 2.2.3. Post-Mortem Examination

After slaughter and evisceration, examination begins with a remote inspection of each carcass and organ. Then, a close inspection is carried out in order to palpate the consistency and to look for the presence of possible lesions by palpation and incision. Each anomaly found is noted in the lesion follow-up register.

#### 2.2.4. Risk Factors

To study risk factors, the species, breed, age, sex and origin of the animal (provenance) and the location, shape, volume, color, consistency and cut surface of the lesion, were recovered and recorded.

In addition, to establish the risk factors involved in the occurrence of pathological findings, the animals were divided and studied according to three age groups (group 1: 1–2 years, group 2: 3–5 years, group 3: 6–8 years).

### 2.3. Statistical Analysis

The data were presented as a percentage and the analysis was carried out using STATISTICA software (Version 10, Stat Soft, 94700 Maison-Alfort, France, 2003). The differences in the results of each parameter were assessed by the Chi-square test. The level of significance was set at *p* < 0.05.

## 3. Results

### 3.1. Overall Results

As shown in the results obtained, out of 1756 cattle slaughtered, 609 (34.68%) presented localized lesions at the level of the different organs with an average of 87.00 ± 90.14 cases (i.e., 4.95 ± 5.13%). In addition, the liver is the most contaminated organ with 227 cases (12.93%) followed by the lungs (184 cases; 10.48%). The lowest frequency was recorded on the digestive system (2 cases; 0.11%) (Table 1) (*p* < 0.001).

### 3.2. Distribution of Lesions According to the Organs Affected

**Figure 1:**
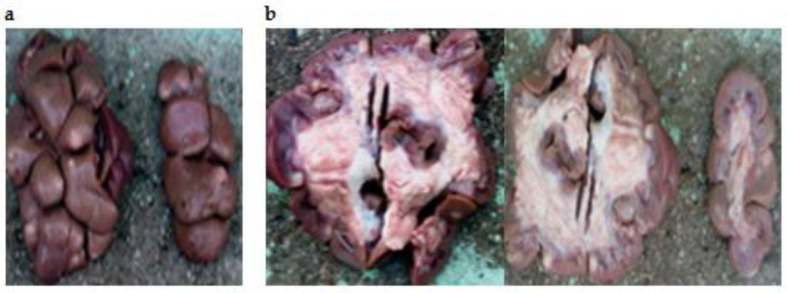
Focal non-purulent interstitial nephritis with whitish foci on the kidney surface (**a**) and on the cross section (**b**).

**Figure 2:**
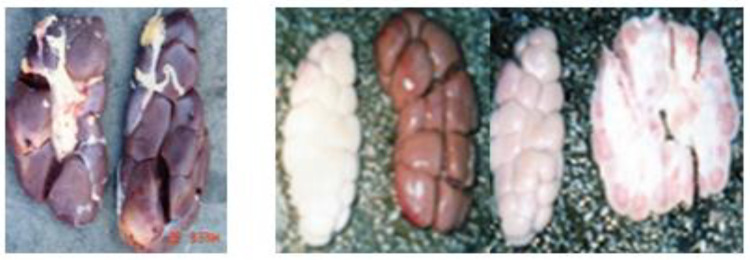
Interstitial nephritis.

**Figure 3:**
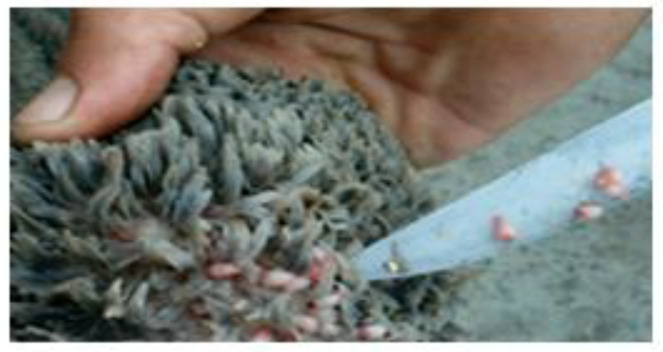
Paramphistomosis.

**Figure 4:**
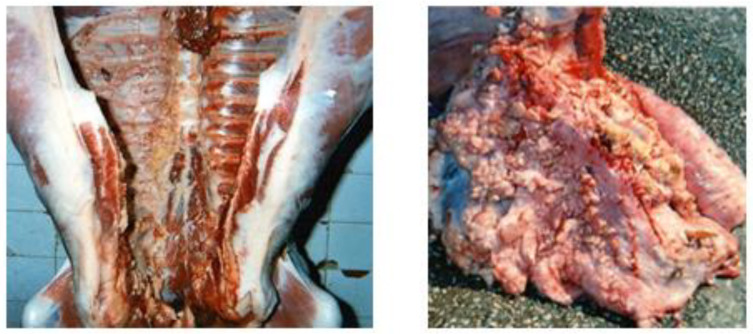
Tuberculosis (Nodularized or miliary appearance with pasty caseiform necrosis).

**Figure 5:**
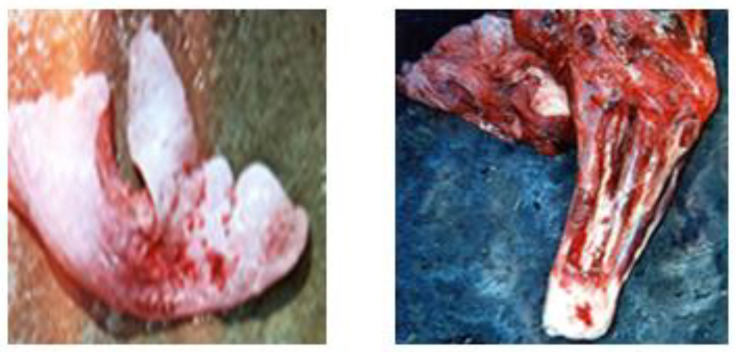
Traumatic carcass (Tissue rupture with hemorrhage).

**Figure 6:**
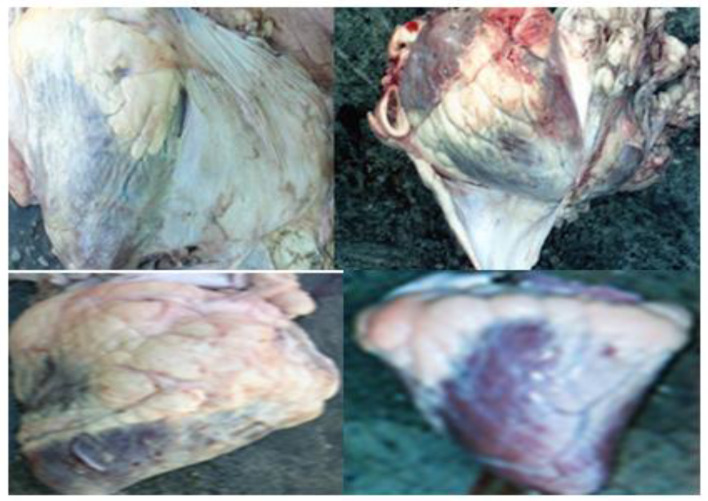
Pericarditis (Pale, sometimes whitish edematous pericardium).

**Figure 7:**
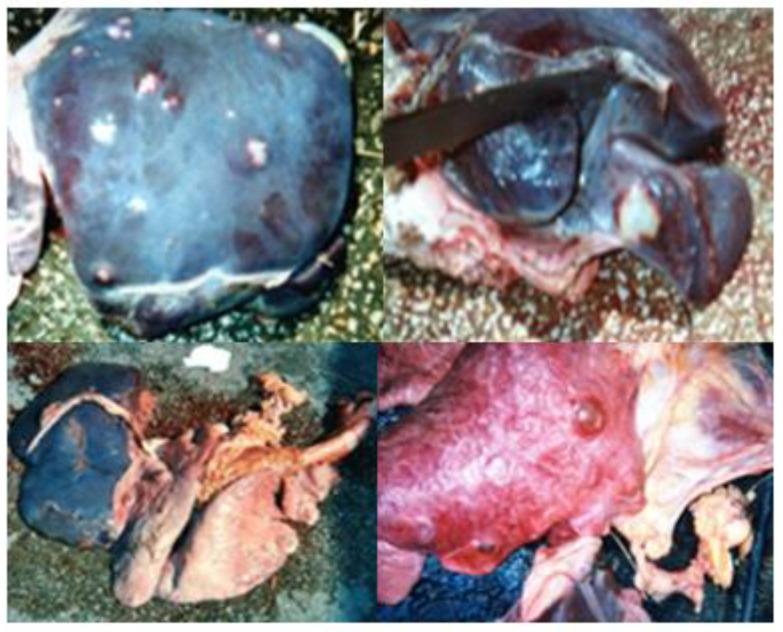
Hydatidosis (Lung and/or liver with a surface full of cysts of variable size with fluid content).

**Figure 8:**
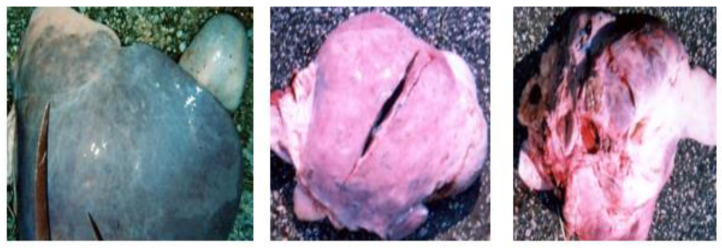
Hepatic lesions.

**Figure 9:**
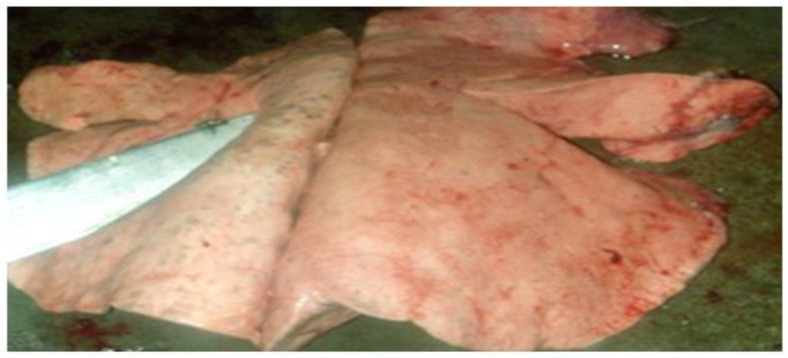
Strongylosis (Candle stains).

The distribution of the cases by organs shows that the highest frequency was recorded on the liver affected by hydatidosis (126 cases; 20.69%), followed by the lungs affected by the same pathological findings (116 cases; 19.05%). While the minimum frequency was revealed on the digestive system affected by paramphistomosis (1 case; 0.16%) and by gastroenteritis (1 case; 0.16%) (Table 2) (Figure 1, Figure 2, Figure 3, Figure 4, Figure 5, Figure 6, Figure 7, Figure 8 and Figure 9).

Analysis of lesion combinations according to the number of abnormalities showed that among the 609 animals with abnormalities, 34 (5.6%) presented only one type of lesion; while 2 (0.3%) showed an association of two types of lesions, the animals presenting three and four types of lesions representing 19.0% and 75.0% of the cases, respectively (*p* < 0.05).

### 3.3. Distribution of Lesions According to the Sex

**Figure 10:**
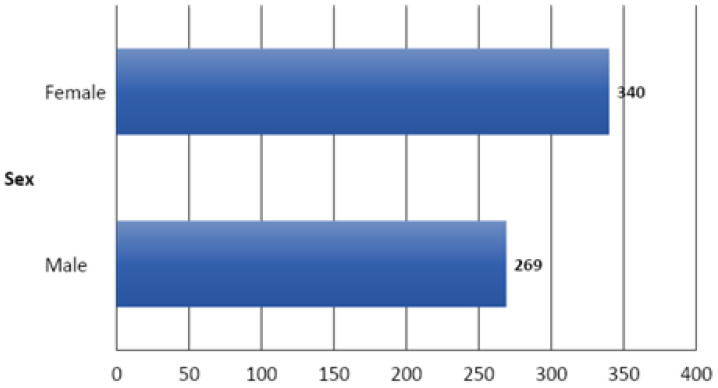
Distribution of lesions according to the sex of the animals.

During the study period, a higher disease frequency was observed in females (55.82%) compared to males (44.17%) (*p* < 0.001) (Figure 10).

### 3.4. Distribution of Lesions by Age Group

**Figure 11:**
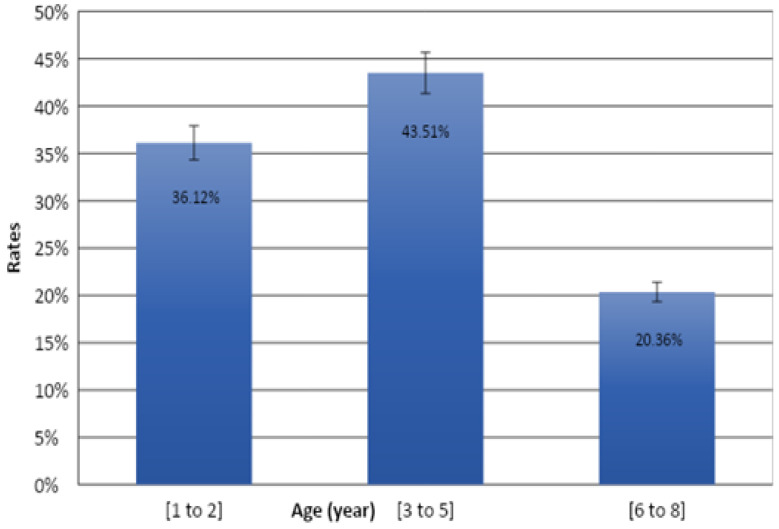
Distribution of lesions by age group.

According to the data, the highest frequency of pathological findings was observed in the age group of 2–5 years (43.51%). The lowest rate was reported in 6–8-year old cattle (20.36%) (*p* < 0.001) (Figure 11).

### 3.5. Distribution of Lesions by Breed

**Figure 12:**
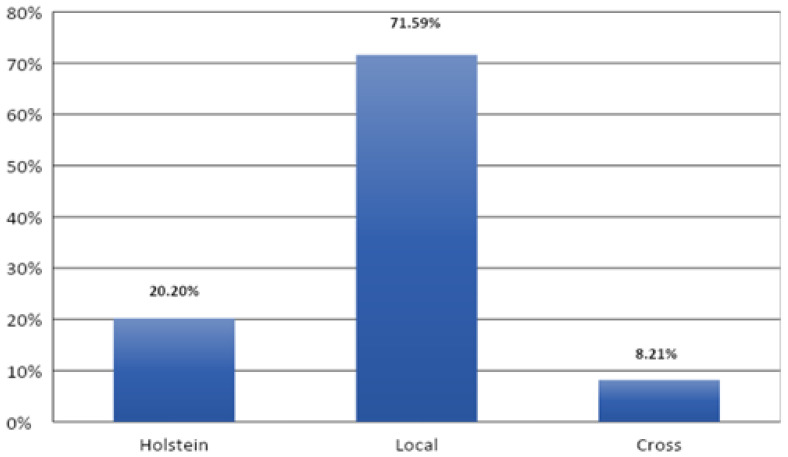
Distribution of lesions by breed.

The distribution of cases by breed showed that local breed cattle are the most affected by pathological findings compared to other breeds, with 436 cases (71.59%). However, there is a clearly significant difference (*p* < 0.001) between disease rate and the races studied (Figure 12).

## 4. Discussion

The main objective of this study was to assess the rate of certain suspicious diseases in the Taher slaughterhouse in the Jijel region in Algeria during a period of fourteen months and to investigate the involvement of some risk factors in the process of the evolution and transmission of these pathological findings and finally to propose corrective measures in order to fight against the diseases.

Data analysis revealed a number of 609 cases for a total of 1756 animals examined. In parallel, a strong presence of certain diseases was noticed as hydatid cyst for the liver and lungs, fasciolosis for the liver and abscesses for the lungs. These results indicate the presence of risk factors favoring the development and transmission of infectious and parasitic agents, which constitutes a major risk for the health of cattle and consumers. In addition, the coastal region of Jijel is considered among the rainiest zones in Algeria. The rainy season lasts around six months, with an annual rainfall of 1103–1252 mm, and records mild temperatures, which creates a sufficiently humid and more temperate microclimate in spite of its Mediterranean type [10]. This may justify the presence of favorable conditions for the survival and transmission of etiological agents and the appearance of diseases thereafter.

Cystic hydatidosis is the most frequent reason for seizure. This lesion corresponds to a larval, inoculable, non-contagious cestodosis common to humans and to various animal species. It is due to the development in the body (particularly in the liver or the lungs) of vesicular larvae of the *Echinococcus* type of parasites living in the adult state in the small intestine of carnivores [11]. There are various species; the most important being *Echinococcus granulosus*. Ithas an indirect life-cycle, with ruminants as intermediate hosts, dogs and other canids as definitive hosts and humans as dead-end hosts [12]. The results of the present study remain clearly superior to those of Kayoueche (2009) [13] in easternAlgeria with a prevalence rate of 9.87%, but they are lower than the results of Belkhiri et al. (2008) [14] in Tiaret and Batna in Algeria with prevalence rates of 26.6%. The latter reported that the hydatid cyst is endemic in the countries of the Mediterranean basin, in particular Algeria. These regions have in common a number of factors which can explain the frequency of this disease, namely the breeding of sheep, the number of stray dogs and the humidity conducive to the maintenance of embryophores in the external environment. Additionally, sometimes there are insufficient hygiene rules; the almost permanent presence of dogs at slaughterhouses, the lack of awareness among breeders and workers and the ineffectiveness of prophylaxis programs. Likewise, the presence of dogs, associated with sheep in family farms and slaughtering during Eid, where the parasitized viscera are distributed to dogs.

Hepatic fascioliasis is a helminthosis particularly affecting ruminants. It is recognized as a parasitic zoonotic worldwide infection. It is due to the development, in the hepatic parenchyma and then in the bile ducts, of helminths which contain two main species; *Fasciola gigantic* and *Fasciola hepatica* [15]. Alongside *Fasciola gigantica*, the *F. hepatica* flukes are widespread trematodes in temperate countries and tropical highlands [16]. It is of great importance because it causes declines in production, and sometimes mortalities and seizures at the level of slaughterhouses. Additional economic losses may be related to expenses on anthelmintic use for treatment and prevention, lower production of milk and wool, reduced weight gain, metabolic diseases and fertility issues [17]. Our results for fascioliasis (11.00% of *Fasciola hepatica*) are significantly lower than those found by Mekroud et al. (2004) [18] with a prevalence rate of 27.0%. On the other hand, they remain superior to the results of Bendiaf (2011) [19], in the region of El Khroub in Constantine in Algeria with a prevalence rate of 7.5%. According to Bentounsi (2001) [20], the nature of the soil (clayey, heavy and smooth soils which favor the development of *Lymnaea*), temperature (no development if the temperature is below 10°C) and humidity can be considered as favoring causes. In addition, according to Hamiroune et al. (2020) [21], The Jijel region is very humid. It is very rich in water resources: five large dams, three large natural lakes and numerous streams; so many indicators of the infestation. In another country, Douttoum et al. (2020) [22] found that the most frequent reason for seizures in N’Djamena (Tchad) was distomatosis (58.91%).

Concerning abscesses, they are diseases that pose a serious risk to the health of cattle and consumers and which are the result of the action of pathogenic agents such as bacteria of the *Staphylococci*, *Pneumococci* or *Streptococci* type which are transmitted by direct inoculation in the tissue. Our results (7.39%) remain clearly superior to those reported by Blaise (2001) [23] in Haiti with a pulmonary prevalence rate of 0.26%. According to Figueroa and Maier (2007) [24] and Malone and Fee (2006) [25], cited by Alloui et al. (2008) [26], several authors state that abscess disease is a pathology found in all intensive farming. At the same time, poor hygiene, precarious shelters and the breeding of several animal species on the same farm seem to be the most determining factors in the appearance of the disease.

Swallowing or hemorrhagic stinging indicates a slaughter defect. This lesion is the result of blood being drawn into the lungs during bleeding. It appears in the form of red spots which are preferentially distributed over the anterior lobes of the lung. Indeed, the lack of sophisticated equipment for slaughter may be the cause of this lesion.

Traumatic meats are characterized by lesions that are generated on farms and just before slaughter by owners and even by those responsible for slaughtering. In addition, they can be the result of the ingestion of a sharp foreign body, capable of crossing the wall of the gastric reservoir and reaching the neighboring organs. They constitute 6.4% of the reasons for seizure. This lesion reflects the negative behavior of breeders and workers by traumatizing animals before slaughter.

In the present work, four cases of pulmonary tuberculosis spread to other organs and tissues (especially the liver and lymph nodes) were recorded. Tuberculosis is a chronic disease mainly caused by bacteria *Mycobacterium bovis* which is a member of the *M. tuberculosis* complex. It is characterized by the progressive development of granulomatous lesions or specific tubercles in the lung tissue, the nodes lymphatics or other organs [27]. In our study, it was a miliary tuberculosis: the size of a grain of millet, with a point of caseification necrosis in its center (caseum). The presence of this zoonosis must be considered a public health problem. Thus, control measures must be strengthened to preserve and to limit the risks related to human and livestock health. A rate of 8.62% was noticed in Tchad for pulmonary and military tuberculosis [22].

Pericarditis was macroscopically expressed by congestion and the presence of fibrin. Adhesions were noted with the pleura and the serosa. The pericardium is very thick (about 3 cm thick). Numerous adhesions associate the pericardium with the parietal pleura on both sides. The tabby-hearted appearance (degenerative myocarditis) characteristic of foot-and-mouth disease has been reported (in this case, only the heart was seized by the veterinary inspector. Pericarditis could be the result of cattle swallowing sharp foreign bodies (metallic or not), causing traumatic reticuloperitonitis complicated to pericarditis. This condition is more frequent in dairy cattle, most often in the last third of gestation or at the time of part [28].

The study of kidney disease is rare compared to diseases of other organs. In this study, kidney lesions were represented by interstitial nephritis resulting from an infection or an allergic reaction to a drug. Our data are in agreement with those found in Iran by Nourmohammadzadeh et al. (2010) [29], who reported that interstitial nephritis was the most common lesion on a total number of 405 cows. Another work performed in Tiaret in southern Algeria by Mahouz et al. (2015) [30] reported high frequencies of kidney diseases (22.66%) in cattle. These authors mentioned interstitial nephritis, glomerulonephritis, epithelionephritis, kidney degeneration and acute tubular necrosis.

A case of bilateral focal non-purulent interstitial nephritis, also called “white spotted kidneys” was noticed in our study. It is a common finding in clinically healthy cattle after slaughter. Although several pathogens can cause this lesion, it is frequently associated with current or prior *Leptospira* spp. infection [29]. This lesion is characterized by irregular whitish areas, of up 1 cm in diameter in the renal cortex which do not normally protrude towards the renal capsule, extend into the parenchyma, and are non-suppurative.

One case of paramphistomosis (rumen fluke disease) was noticed in the current work. This trematodosis is due to several genera such as Calicophoron, Cotylophoron, Explanatum, Gigantocotyle and Paramphistomum [31]. Titi et al. (2014) [10] determined the seasonal variations in the prevalence and intensity of bovine paramphistomosis in a Mediterranean climate and identified paramphistome species using molecular biology from abattoir specimens in Jijel. According to Meguini et al. (2021) [16], paramphistomosis and fasciolosis are considered two of the major parasitic diseases in northeastern Algeria, sharing the same intermediate host (*G. truncatula*), which makes the epidemiology of diseases caused by these two parasites quite close to each other.

In this study, the most encountered lesion in the genital tract was uterine infection followed by ovarian cyst and inflammatory salpinx; this result proved that contamination and infection are always present in the cowsheds from where animals were brought. These infections result from the *Arcanobacterium pyogenes*, either alone or together with other pathogenic microorganisms such as: *Fusobacterium necrophorum*, *Bacteroides* spp. or *Escherichia coli*. Data are in agreement with those reported in previous works [32,33].

Likewise, environmental factors play a key role in the appearance, distribution and evolution of the pathological findings studied, which can be caused by different factors that are generally closely associated. Only stress factors can induce a disease and they will always influence, associated with virulent or non-virulent agents, the appearance and the gravity of the situation. Climatological variations, whatever their nature, also play a role. Changes in temperature, humidity and winds, extreme events, vegetation as a whole and landscapes in general favor epidemiological variations in infectious diseases, their emergence, their expression, their severity, their possibility scalable, etc. Warming is a fundamental source of changes in the multiplication of germs, the establishment or disappearance of others, directly or through variations in reservoirs or transmission vectors [34].

The distribution of results by sex and race showed that females of local breed are more affected by diseases compared to males of other breeds. These results can be explained by several factors, in particular, during the fattening period, in the majority of cases, the animals (mainly the males) prepared for slaughter, are raised in an intensive system. In addition, depending on the ability of breeders and practicing veterinarians who monitor the breeding, these animals are treated with antiparasitics against parasites and with antibiotics against infectious diseases, as well as other drugs like vitamins, which leads to a decrease in the number of cases of parasitized organs despite the presence of humidity in the external environment [21]. Besides, in the region of Jijel, sheep farms, especially local breeds (mainly females) are reared extensively (unlike other breeds) which promotes the transmission of various infectious diseases in the case of the presence of survival factors and the development of contamination agents. Moreover, the sacrificed males are generally young and very vigorous.

In addition, the present study shows that the overall disease rate is higher in cattle aged 2–5 years. It should be noted that in Algeria, animals of this age category are the most slaughtered. Furthermore, there can be females in the case of wedding parties, for example. At the same time, these results can be explained by the decrease in immunity and the exposure of this age group to infectious and parasitic diseases as well as the high number of slaughter animals of this category group. In parallel, in young animals (1 to 2 years old) the infestations are strong and the disorders more serious and that acquired immunity sets in with age on the one hand, and contact with the parasite on the other. In addition, this rate of disease is higher than that observed in animals aged 6 to 8 years. This can be explained by the fact that ruminants develop resistance to pathogens linked to repeated infestations with age [35].

Finally, it is interesting to know the prevalence of pathological findings in animals slaughtered in Algerian slaughterhouses, because they cause significant economic losses in meat and constitute a major risk for the health of consumers. It is important to notice that there is a need to call for further application of improved control and prevention measures. It is recommended to apply preventive treatments regularly in order to reduce the number of positive cases and to eradicate the pathology thereafter.

## 5. Conclusions

Our data confirm the importance of the bovine diseases requiring research in Algerian slaughterhouses such as hydatidosis, fascioliasis and abscesses. The detection of these diseases is linked to the presence of infectious or non-infectious agents, which constitutes a risk to the health of cattle and consumers. In addition, the present pathological findings are associated with some factors. It predominantly affects female cattle, from local breed, aged 3 to 5 years. At the same time, the results obtained make it possible to discover the risks of collective food poisoning in the event of the consumption of offal and half-cooked grilled meats, in particular, with the evolution of Algerian food practices and the development of the fast-food sector. Finally, the eradication of the agents responsible for these diseases remains essential for the protection of the health of consumers. It is important to act on the life cycles of the pathogens inducing these diseases in order to control the transmission of the causative agents upstream, either, for example, on one hand, by preventing the access of dogs to livestock carcasses, by treating dogs with a vermifuge and vaccinating animals in the case of a hydatid cyst, or on the other hand, the fight against the intermediate host, the correct arrangement of the pits, the detection of the parasite in the environment by the detection molluscs and the prohibition of any consumption in the raw state of plants taken from the natural environment, in the event of fascioliasis. Furthermore, medical prophylaxis remains an important act in the fight against the diseases studied. In addition, in the laboratory, strict hygiene is necessary to avoid infestation of personnel. In addition, sensitization and popularization of farmers, consumers and other actors in the food chain are mandatory.

Likewise, it will be very useful to carry out systematic screening in all farms in the country through general and specific laboratory tests for confirmation. For example, the tuberculin test remains the most effective in Algeria for the diagnosis of bovine tuberculosis. It is a test applicable to male and female cattle from the age of six months. It is mandatory for cattle farms where the breeder has an agreement with the dairies, in the event of an epidemic and in the event of the discovery of lesions at the slaughterhouse, and the performance of tuberculinization tests in the farm of origin of the sick animal is obligatory. All of this will contribute to a better fight against diseases, but would also make it possible to improve the independence of Algeria vis-à-vis countries in terms of importing meat, thus ultimately providing benefits for the entire economy of the country.

## Figures and Tables

**Table 1:** Overall data concerning the number of positive cases.

Organs	Number of Cases	%
Lung	184	10.48
Liver	227	12.93
Carcass	46	2.62
Heart	27	1.54
Kidney	7	0.40
Digestive system	2	0.11
Reproductive system	116	6.61
Total	609	34.68
Mean ± SD	87.00 ± 90.14	4.95 ± 5.13
*p* value		<0.001

**Table 2:** Distribution of lesions according to the organs affected.

Organ	Lesion	Number of Cases	%	*p* Value
Lung	Slaughter defect	11	1.81	*p* < 0.05
	Pneumonia	12	1.97	
	Abscess	45	7.39	
	Hydatidosis	116	19.05	
Liver	Fascioliasis	67	11.00	*p* < 0.05
	Hepatitis	28	4.60	
	Hydatidosis	126	20.69	
	Telangiectasia	6	0.98	
Carcass	Traumatic meat	39	6.40	*p* < 0.05
	Generalized tuberculosis	4	0.66	
	Jaundice	2	0.33	
	Others	1	0.16	
Heart	Pericarditis	27	4.43	
Kidney	Nephritis	7	1.15	
Digestive	Paramphistomosis	1	0.16	*p* > 0.05
	Gastroenteritis	1	0.16	
Urogenital system	Uterine infections	53	8.70	*p* < 0.05
	Ovarian cyst	41	6.73	
	Salpingitis	22	3.61	
Total		609	99.99	
Mean ± SD		30.45 ± 36.75	5.00 ± 6.04	

## Data Availability

The data presented in this study are available on request from the corresponding authors.

## References

[1] Godfray H., Charles J., Aveyard P., Garnett T., Hall J.W., Key T.J., Lorimer J., Pierrehumbert R.T., Scarborough P., Springmann M. (2018). Meat consumption, health, and the environment. Science.

[2] APS (2017). Viandes Rouges: Une Production Nationale de Plus de 5 Millions de Quintaux en 2017. https://www.aps.dz/economie/75838-viandes-rouges-une-production-nationale-de-plus-de-5-millions-de-quintaux-en-2017.

[3] APS (2021). Gel des Importations des Viandes Rouges: 200 Millions USD à Préserver Annuellement. https://www.aps.dz/economie/116361-importation-des-viandes-rouges-plus-de-200-millions-de-dollars-a-preserver-annuellement.

[4] Williams P., Brent P., Mann J., Truswell A.S. (2017). Essentials of Human Nutrition.

[5] Mimoune N., Seddiki S., Baazizi R., Saboundji I.E., Saidi R., Khelef D., Kaidi R. (2021). Antibiotic residues in cow’s milk. Vet. Stanica.

[6] Mimoune N., Saidi R., Benadjel O., Khelef D., Kaidi R. (2021). Alternative treatment of bovine mastitis. Vet. Stanica.

[7] Lamari I., Mimoune N., Khelef D. (2021). Effect of feed additive supplementation on bovine subclinical mastitis. Vet. Stanica.

[8] de Jijel W. (2011). Rubrique Monographie Wilaya.

[9] Rodier J., Legube B., Merlet N., Brunet R., Nafa S., Hamrouche A. (2009). L’analyse de l’eau. Eau naturelles, Eaux Résiduaires, eau de mer. Caractérisation des Eaux D’irrigation Destinées à L’agriculture Dans le Périmètre de Jijel-Taher.

[10] Titi A., Mekroud A., Chibat M.E.H., Boucheikhchoukh M., Zein-Eddine R., Djuikwo-Teukeng F.F., Vignoles P., Rondelaud D., Dreyfuss G. (2014). Ruminal paramphistomosis in cattle from northeastern Algeria: Prevalence, parasite burdens and species identification. Parasite.

[11] Ibrahem M.M., Ibrahem W.M., Abdorrahem M.M., Ibrahem K.M. (2016). Livestock Hydatid Disease (Cystic Hydatidosis) in Libya: A Review. Am. J. Anim. Vet. Sci..

[12] Stoore C., Andrade C., Hidalgo C., Corrêa F., Jiménez M., Hernandez M., Paredes R. (2018). *Echinococcus granulosus* hydatid cyst location is modified by *Fasciola hepatica* infection in cattle. Parasites Vectors.

[13] Kayoueche F.Z. (2009). Epidemiology of Hydatidosis and Fascioliasis in Animals and Humans in Eastern Algeria. Ph.D. Thesis.

[14] Belkhiri M., Tlidjane M., Meziane T. (2008). Fréquence des lésions pulmonaires des bovins et ovins de Tiaret et Batna (Algérie). Frequency of sheep and cattle lung lesions in Tiaret and Batna (Algeria). Département vétérinaire—faculté des sciences—université de Batna—Algérie. Renc. Rech. Rumin..

[15] Elshraway N.T., Mahmoud W.G. (2017). Prevalence of fascioliasis (liver flukes) infection in cattle and buffaloes slaughtered at the municipal abattoir of El-Kharga, Egypt. Vet. World.

[16] Meguini M.N., Righi S., Bouchekhchoukh M., Sedraoui S., Benakhla A. (2021). Investigation of flukes (*Fasciola hepatica* and *Paramphistomum* sp.) parasites of cattle in north-eastern Algeria. Ann. Parasitol..

[17] Nyirenda S.S., Sakala M., Moonde L., Kayesa E., Fandamu P., Banda F., Sinkala Y. (2019). Prevalence of bovine fascioliasis and economic impact associated with liver condemnation in abattoirs in Mongu district of Zambia. BMC Vet. Res..

[18] Mekroud A., Benakhla A., Vignoles P., Rondelaud D., Dreyfuss G. (2004). Preliminary studies on the prevalences of natural fasciolosis in cattle, sheep, and the host snail (*Galba truncatula*) in north-eastern Algeria. Parasitol. Res..

[19] Bendiaf H. (2011). Contribution à L’étude de la Distomatose à *Fasciola hepatica* (*Linné,* 1758): Aspects Parasitologique et Sérologique. Master’s Thesis.

[20] Bentounsi B., Mecif A., Kohil K. (2001). Evolution du parasitisme ovin sur un élevage de la région du Khroub. Approche par les méthodes coproscopiques. Sci. Technol..

[21] Hamiroune M., Dahmane M., Charef A., Cheniguel H., Foughalia H., Saidani K., Djemal M. (2020). Evaluation of Fascioliasis, Hydatidosis, and Tuberculosis in Domestic Animals during Post-Mortem Inspection at Jijel Slaughterhouse (Algeria). J. Food Qual. Hazards Control.

[22] Doutoum A.A., Hamid A.A., Doungous D.M., Sakhaïroun A., Tidjani A., Markhous A.N., Moukhtar R., Seydi M., Abdourahamane B. (2020). Motifs de saisies de viandes rencontrées à l’abattoir frigorifique de Farcha (N’Djamena/Tchad). Rev. Sci. Du Tchad Série B-Janvier.

[23] Blaise F. (2001). Prévalence et fréquence des lésions parasitaires du foie et du poumon des ruminants en Haïti. Rev. Méd. Vét..

[24] Figuerora J.P., Maier N.L. (2007). Veme Congreso de Especialistas en pequenos ruminantes y camelidos Sudamericanos, Mendoza, Argentina. https://www.uchile.cl/agenda/121542/x-congreso-latinoamericano-en-pequenos-rumiantes-y-camelidos.

[25] Malone F.E., Fee S.A. (2006). A serological investigation of caseous lymphadenitis in four flocks of sheep. Irish Vet. J..

[26] Alloui M.N., Ayachi A., Alloui N., Tlidjane M., Kaba J. (2008). Prévalence de la maladie des abcès des petits ruminants dans la región de Batna (Algérie). Renc. Rech. Rumin..

[27] Benet J.J., Praud A. (2014). La Tuberculose Animale.

[28] Nielsen S.S., Denwood M.J., Forkman B., Houe H. (2017). Selection of Meat Inspection Data for an Animal Welfare Index in Cattle and Pigs in Denmark. Animals.

[29] Nour mohammadzadeh F., Haji Hajikolaei M.R., Sasani F., Alidadi N. (2010). Abattoir study of the prevalence of renal lesions in slaughtered cattle. Int. J. Vet. Res..

[30] Mahouz F., Khoudja F.B., Chikhaoui M. (2015). Pathological study on renal Diseases in Cattle and Sheep. J. Anim. Vet. Adv..

[31] Kilani M., Chermette R., Guillot J., Polack B., Duncan J.L., Cabaret J., Lefèvre P.C., Blancou J., Chermette R., Uilenberg G. (2010). Gastrointestinal helminthoses: Amphistomosis. Infectious and Parasitic Diseases of Livestock.

[32] Mimoune N., Kaidi R., Azzouz M.Y., Keddour R., Belarbi A., Derdour S.Y. (2016). Genital Tract Pathologies of Cows Slaughtered at El-Harrach Abattoir in Algeria. Kafkas Univ. Vet. Fak. Derg..

[33] Mimoune N., Khelef D., Kaidi R. (2021). Ovarian cysts in cattle: A survey among veterinary practitioners in Algeria. Vet. Stanica.

[34] Escobar L.E., Romero-Alvarez D., Leon R., Lepe-Lopez M.A., Craft M.E., Borbor-Cordova M.J., Svenning J.-C. (2016). Declining Prevalence of Disease Vectors under Climate Change. Sci. Rep..

[35] Doyle J.J. (1972). Evidence of an acquired resistance in calves to a single experimental Infection with *Fasciola hepatica*. Res. Vet. Sci..

